# Geometrical Comparison and Quantitative Evaluation of ^18^F-FDG PET/CT- and DW-MRI-Based Target Delineation Before and During Radiotherapy for Esophageal Squamous Carcinoma

**DOI:** 10.3389/fonc.2021.772428

**Published:** 2021-12-22

**Authors:** Huimin Li, Jianbin Li, Fengxiang Li, Yingjie Zhang, Yankang Li, Yanluan Guo, Liang Xu

**Affiliations:** ^1^ Weifang Medical University, Weifang, China; ^2^ Department of Respiratory and Neurology, The Affiliated Tumor Hospital of Xinjiang Medical University, Urumqi, China; ^3^ Department of Radiation Oncology, Shandong Cancer Hospital and Institute, Shandong First Medical University and Shandong Academy of Medical Sciences, Jinan, China; ^4^ Department of Positron Emission Tomography-Computed Tomograph (PET-CT), Shandong Cancer Hospital and Institute, Shandong First Medical University and Shandong Academy of Medical Sciences, Jinan, China; ^5^ Department of Medical Imaging, Shandong Cancer Hospital and Institute, Shandong First Medical University and Shandong Academy of Medical Sciences, Jinan, China

**Keywords:** esophageal squamous carcinoma, diffusion magnetic resonance imaging, positron emission tomography, gross target volume, standard uptake value (SUV), apparent diffusion coefficient (ADC)

## Abstract

**Background and Purpose:**

This study aimed to evaluate the geometrical differences in and metabolic parameters of ^18^F-fluorodeoxyglucose positron emission tomography–computed tomography (^18^F-FDG PET-CT) and diffusion-weighted magnetic resonance imaging (DW-MRI) performed before and during radiotherapy (RT) for patients with esophageal cancer based on the three-dimensional CT (3DCT) medium and explore whether the high signal area derived from DW-MRI can be used as a tool for an individualized definition of the volume in need of dose escalation for esophageal squamous cancer.

**Materials and Methods:**

Thirty-two patients with esophageal squamous cancer sequentially underwent repeated 3DCT, ^18^F-FDG PET-CT, and enhanced MRI before the initiation of RT and after the 15th fraction. All images were fused with 3DCT images through deformable registration. The gross tumor volume (GTV) was delineated based on PET Edge on the first and second PET-CT images and defined as GTV_PETpre_ and GTV_PETdur_, respectively. GTV_DWIpre_ and GTV_DWIdur_ were delineated on the first and second DWI and corresponding T_2_-weighted MRI (T_2_W-MRI)-fused images. The maximum, mean, and peak standardized uptake values (SUVs; SUV_max_, SUV_mean_, and SUV_peak_, respectively); metabolic tumor volume (MTV); and total lesion glycolysis(TLG) and its relative changes were calculated automatically on PET. Similarly, the minimum and mean apparent diffusion coefficient (ADC; ADC_min_ and ADC_mean_) and its relative changes were measured manually using ADC maps.

**Results:**

The volume of GTV_CT_ exhibited a significant positive correlation with that of GTV_PET_ and GTV_DWI_ (both *p* < 0.001). Significant differences were observed in both ADCs and ^18^F-FDG PET metabolic parameters before and during RT (both *p* < 0.001). No significant correlation was observed between SUVs and ADCs before and during RT (*p* = 0.072–0.944) and between ∆ADCs and ∆SUVs (*p* = 0.238–0.854). The conformity index and degree of inclusion of GTV_PETpre_ to GTV_DWIpre_ were significantly higher than those of GTV_PETdur_ to GTV_DWIdur_ (both *p* < 0.001). The maximum diameter shrinkage rate (∆LD_DWI_) (24%) and the tumor volume shrinkage rate (VRR_DWI_) (60%) based on DW-MRI during RT were significantly greater than the corresponding PET-based ∆LD_PET_ (14%) and VRR_PET_ (41%) rates (*p* = 0.017 and 0.000, respectively).

**Conclusion:**

Based on the medium of CT images, there are significant differences in spatial position, biometabolic characteristics, and the tumor shrinkage rate for GTVs derived from ^18^F-FDG PET-CT and DW-MRI before and during RT for esophageal squamous cancer. Further studies are needed to determine if DW-MRI will be used as tool for an individualized definition of the volume in need of dose escalation.

## Introduction

Radiotherapy—one of the main effective and relatively safe treatment modalities—is now fully integrated in the multidisciplinary treatment of esophageal cancer (EC). Currently, with substantial evidence, radiotherapy can be applied as a sole treatment or as part of a comprehensive treatment in combination with systemic treatments such as surgery, chemotherapy, targeted therapy, and, more recently, immunotherapy (1). Regional recurrence accounts for most radiation treatment failures in EC cases, with a local relapse rate of 40% (2). In particular, 90% of locoregional failures after definitive chemoradiotherapy (dCRT) occurred within the gross tumor volume (GTV) (3). Hence, there is an urgent need to escalate the radiation dose to the area at highest risk of recurrence to improve locoregional control. Currently, there is a growing interest in the delivery of intensity-modulated radiotherapy (IMRT)-based late course boost or simultaneously integrated boost techniques (4, 5), which could selectively deliver high radiation doses to radioresistant regions and a relatively low dose to subclinical tissues.

Currently, metabolic and functional imaging modalities such as ^18^F-fluorodeoxyglucose positron emission tomography–computed tomography (^18^F-FDG PET-CT) and diffusion-weighted magnetic resonance imaging (DW-MRI) are gaining increasing clinical significance in the management of patients undergoing radiotherapy since these allow visualization and quantification of treatment-induced changes on a molecular level before volumetric changes become apparent (6–8). It is well known that PET-based parameters such as standardized uptake value (SUV), metabolic tumor volume (MTV), and total lesion glycolysis (TLG) have been established and validated as prognostic biomarkers in EC (8, 9). Escalating the radiation dose to 64.8 Gy, which had been previously established, failed to improve survival or locoregional control (10). It is warranted to explore potential tools for an individualized definition of the volume in need of dose escalation. The current analysis demonstrates that high FDG uptake on initial PET-CT can identify tumor areas at high risk of relapse in EC (9, 11). Another study by Yu et al. (5) showed that the FDG hotspot within the residual area was completely within the GTV and remained stable during RT. They also reported that adaptive RT based on target volume reduction assessed on PET-CT could facilitate dose escalation up to 70 Gy, with a 1-year overall survival and local control of 69.2% and 77.4%, respectively. Therefore, it is feasible and safe to select boosting of high ^18^F-FDG uptake zones within the tumor based on FDG PET-CT for the definition of the volume in need of dose escalation.

However, repeated PET imaging has not been widely adopted regardless of its clinical benefit owing to radiation exposure and uncertain segmentation algorithms obtained during PET (12, 13). In contrast, considering patient acceptability, repeated MRI is generally well tolerated for response assessment (14). High-resolution MRI for target volume delineation and response assessment in EC is currently of immense clinical interest (15, 16). The apparent diffusion coefficient (ADC) map from DW-MRI is a quantitative measure for the motion of water molecules and inversely correlates with tissue density. Relevant studies have shown that lower ADC values were associated with a higher histological grade and aggressiveness (17). Furthermore, it has been recently recognized that relative ADC changes from baseline to interim DW-MRI scans can help identify pathologic response in EC patients (7). Hence, we could theoretically observe the feasibility of selective boosting of the high signal areas of EC based on DW-MRI for definition of the volume in need of dose escalation.

Currently, selective boosting of high ^18^F-FDG uptake zones based on FDG PET-CT within the tumor has been suggested for radioresistance (5, 9, 11). To date, CT imaging of the tumor extension remains the gold standard for target volume contouring and plan evaluation. Therefore, based on the medium of CT images, we evaluated the spatial position and functional parameters of ^18^F-FDG PET-CT and DW-MRI performed before and during radiotherapy in patients with esophageal squamous carcinoma. The aim of this study was to explore whether the high signal area derived from DW-MRI can be used as tool for an individualized definition of the volume in need of dose escalation for esophageal squamous cancer.

## Materials and Methods

### Patient Selection and Characteristics

After receiving approval from the local research ethics committee, a total of 35 patients with newly diagnosed, biopsy-proven, nonmetastatic esophageal squamous cancer suitable for concurrent chemoradiotherapy were recruited for this prospective study between November 2016 and May 2020. All patients scheduled to receive neoadjuvant or definitive chemoradiation for EC underwent 3DCT, ^18^F-FDG PET-CT, and MRI simulation scanning prior to the initiation of RT and after 15 fractions of RT. Written informed consent was obtained from every patient included in this study. Patients were excluded if either pre-RT ^18^F-FDG PET-CT or DW-MRI data were not available (*n* = 1), the volume of the tumor on baseline metabolic imaging was extremely small (≤1 cm^3^) (*n* = 1), or they did not complete RT (*n* = 1). Consequently, image data of 32 patients were available for analysis. Patient and treatment characteristics are presented in **Table 1**.

**Table 1:** Patient and treatment characteristics.

	Number	Percent
**Patient characteristics**		
Age (year), median (range)	67	(47–76)
Sex		
Female	6	18.8
Male	26	81.3
ECOG PS		
0–1	32	100.0
2	0	0.0
Pathology		
Squamous cell carcinoma	32	100.0
Adenocarcinoma	0	0.0
Site^a^		
Upper thoracic (UI 20–25 cm)	12	37.5
Middle thoracic (UI 25–30 cm)	11	34.4
Lower thoracic (UI 30–40 cm)	9	28.1
Stage^b^		
II	3	9.3
IIIA	4	12.5
IIIB	23	71.9
IVA	3	9.3
**Treatment characteristics**		
Aim		
Definitive chemoradiation	28	87.5
Neoadjuvant chemoradiation	4	12.5
Chemotherapy regimen		
5-Fluorouracil+cisplatin	30	93.8
5-Fluorouracil monotherapy	2	6.2
RT modality		
IMRT	32	100.0
3D-CRT	0	0.0
Total dose (Gy), median (range)	60	(41.4-60)
Fraction dose (Gy), median (range)	2.0	(1.8-2.0)
Fractions of RT completed before midradiotherapy PET/DWI (fractions)	15	100.00
Dose of RT completed before midradiotherapy PET/DWI (Gy), median (range)	30	(27.30)

ECOG, PS Eastern Cooperative Oncology Group performance status; UI, upper incisor; RT, radiotherapy; IMRT, intensity-modulated radiotherapy; 3D-CRT, 3-dimensional conformal radiotherapy.

a American Joint Committee on Cancer classification 2017.

b Clinical tumor-node-metastasis (cTNM) stage according to 8th edition TNM classification.

### Image Simulation and Acquisition

Each patient underwent contrast-enhanced CT using a 16-slice CT scanner (Philips Brilliance Bores CT, Cleveland, OH, USA), with a 3-mm slice thickness during free breathing. All patients were scanned in the supine position, followed by laser alignment. The ^18^F-FDG PET–CT examinations were performed within 2 weeks before the initiation of RT (PET_pre_) and after 15 fractions (median 27 Gy, 1.8 Gy per fraction) of RT (PET_dur_). Following CT, PET_pre_ was performed from the proximal thigh to the base of the skull in 3D acquisition mode with 2–5 min per bed position, while PET_dur_ was acquired from the skull base to the diaphragm. PET images were reconstructed using iterative 3D reconstruction.

Patients underwent MRI scanning with anatomical (T_2_-weighted) and functional (diffusion-weighted) MRI sequences at the same two time points as that for ^18^F-FDG PET-CT. MRI examinations were performed on a 3.0-T scanner equipped with a 32-tunnel body phased-array coil (Discovery MR 750, GE Medical Systems, Milwaukee, WI, USA). Patients were scanned in the supine position, with arms parallel to the body for both pre- and mid-RT scanning. Transverse DW images were obtained under free breathing conditions with the following scan parameters: repetition time (TR) 13,333 ms, echo time (TE) 64 ms, acquisition matrix 128 × 128 mm, field of view (FOV) 500 × 500 mm, slice thickness = 3.6 mm, and NE*_X_* 5. A diffusion-sensitive gradient *b*-value of 600 s/mm^2^ was applied for DWI. T_2_W-MRI adopts fast spin echo to scan cross and axial sections, with the following specific parameters: TR 12,000 ms, TE 84 ms, thickness and spacing 3 mm, FOV 500 × 500 mm, acquisition matrix 384 × 384, and NE*_X_* 1.8. Additionally, conventional T_2_W-MR images were obtained using pulse and respiratory gating techniques to trigger scanning exclusively during the end of expiration (18).

### Image Registration and Target Delineation

The ^18^F-FDG PET-CT and MR images were registered to the planning CT using deformable image registration (DIR) in the software MIM Vista^®^ (MIM Software Inc., version 6.8.3, Cleveland, OH, USA). The main role of DIR is to define spatial correspondence between two considered image sets. To ensure the accuracy and repeatability of the delineation of target volumes, all structures were delineated by the same experienced radiation oncologist according to the consensus guidelines. GTVs were manually contoured on the first and second planning CT images, referred to as GTV_CTpre_ and GTV_CTdur_, with a mediastinal window (window width = 400 HU, window level = 40 HU) setting and by the following standards: the GTVs were defined as any enlargement of the esophagus over its standard dimensions, 5 mm for wall thickness and 10 mm for wall diameter. On the basis of the reconstructed ^18^F-FDG PET-CT image, given that no single absolute and relative methods of PET-based target volume delineation were validated, a gradient-based segmentation algorithm (PET_Edge_) was applied, which identified tumors on the basis of changes in intensity/activity concentration at the tumor borders (19). The GTVs based on the first and second PET-CT images were determined using thresholds of PET_Edge_ and defined as GTV_PETpre_ and GTV_PETdur_, respectively. All noncancerous regions within the GTV_PET_, including areas overlaid by the heart, bone, and great vessels, were corrected to be excluded manually with the help of the CT component of PET-CT. Similarly, GTV_DWIpre_ and GTV_DWIdur_ were delineated on the first and second DWI and corresponding T_2_-weighted MRI-fused images.

### Functional Parameter Extraction

Images were analyzed and measured by two observers (a senior radiologist and an imaging physician in diagnostic PET/MRI) who were blinded to the histopathological results. Regions of interest (ROIs) were automatically drawn on the first and second PET based on PET_Edge_ and were modified to exclude any overlap with the heart, bone, and great vessels on CT images of PET-CT. The maximum, mean, and peak standardized uptake values (SUV_max_, SUV_mean_, and SUV_peak_, respectively), MTV, and TLG were calculated automatically using the MIM software. TLG is defined as MTV from PET multiplied by SUV_mean_ within that volume (20).

For ADC measurements, an ADC map in grayscale was automatically generated in DW-MRI using ADW 4.7 Workstation (GE Healthcare, Waukesha, WI, USA). The manifested largest and clearest sections of esophageal lesions were selected as the ROIs. Subsequently, ROIs on the DWI and corresponding T_2_-weighted MRI fused images were edited manually by two physicians in consensus to ensure that areas of hemorrhage, necrosis, edema, cystic change, and normal vessels were excluded. Finally, through the MIM software, the positions of ROIs on the ADC map were set to the same layers and locations prior to RT and after the 15th treatment cycle. The mean and minimum ADCs (ADC_mean_ and ADC_min_, respectively) of the lesion were automatically calculated. The relative changes in percent (∆%) of these ^18^F-FDG PET-CT and DW-MRI parameters (i.e., ∆ADCs, ∆SUVs, ∆MTV, and ∆TLG) between baseline scans and scans during RT were calculated.

### Overlap Analysis

To quantify the overlap between PET-CT- and DW-MRI-based delineations before and during RT, the conformity index (CI) and degree of inclusion (DI) were calculated for GTV_PETpre_ and GTV_DWIpre_ and GTV_PETdur_ and GTV_DWIdur_, respectively. The CI of volume *A* and *B* (CI[*A*, *B*]) was computed according to that described in a study by Struikmans et al. (21). A CI of 1 indicates 100% agreement between GTVs, and a CI of 0 indicates no overlap in delineation. The formula was as follows:


$$\text{CI}(A,\;B)=\frac{A\cap B}{A\cup B}$$

The definition of DI of volume *A* included in volume *B* (DI[*A* in *B*]) was the intersection between volume *A* and volume *B* divided by volume *A* (22). The DI was defined as follows:


$$\text{DI}(A\;\text{in}\;B)=\frac{A\cap B}{A}$$

### Tumor Shrinkage Analysis

Based on the Response Evaluation Criteria in Solid Tumors version 1.1, measurements of tumor volume and tumor maximal diameters were performed on PET-CT or MR images prior to and during RT. Four diameters measured on the first and second PET and DWI images were defined as LD_PETpre_ and LD_PETdur_ and LD_DWIpre_ and LD_DWIdur_, respectively. The percentage maximum diameter shrinkage rate (∆LD) was calculated using the following equation:


$$\Delta \text{LD}=\frac{\left[(\text{L}{\text{D}}_{\text{pre}}-\text{L}{\text{D}}_{\text{dur}})\right]}{\text{L}{\text{D}}_{\text{pre}}}\times 100\%$$

By the same method, the volume reduction rate (VRR) was calculated as follows:


$$\text{VRR}=\frac{\left([\text{GTVpre}]-[\text{GTVdur}]\right)}{\text{GTVpre}}\times 100\%$$

### Statistical Analysis

Statistical analysis was performed using SPSS 21.0 software (IBM Corp, Armonk, NY, USA). Data with skewed distribution are presented as medians with ranges. The Wilcoxon signed-rank test was used to compare the target volumes and relevant parameters. Spearman’s rank correlation analysis was performed to analyze the relativity between SUVs and ADCs. A *p*-value <0.05 indicated statistical significance.

## Results

### Correlation Analysis of GTVs and GTV_CT_

**Figure 1:**
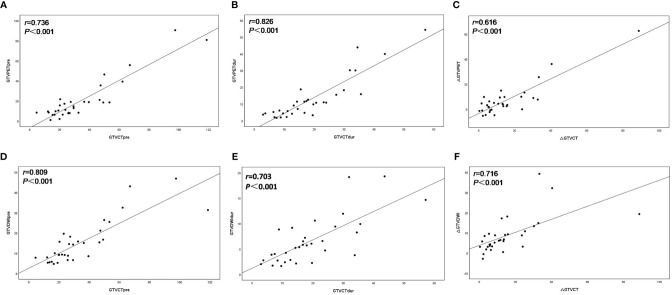
Scatter plots of correlation between the target volume delineated on^18^F-FDG PET–CT (GTV_PET_) and DW-MRI (GTV_DWI_) and on the corresponding CT (GTV_CT_) before and during radiotherapy. The best-fit line is shown as the solid line for each scatterplot. **(A)** GTV_PETpre_ versus GTV_CTpre_; **(B)** GTV_PETdur_ versus GTV_CTdur_; **(C)** ΔGTV_PET_ versus ΔGTV_CT_; **(D)** GTV_DWIpre_ versus GTV_CTpre_; **(E)** GTV_DWIdur_ versus GTV_CTdur_; **(F)** ΔGTV_DWI_ versus ΔGTV_CT_.

The volume of GTV_CTpre_ was 26.34 (4.50–118.71) cm^3^, leading to a significantly positive correlation with both GTV_PETpre_ (**Figure 1A**) and GTV_DWIpre_ (**Figure 1D**) (*r* = 0.763 and *r* = 0.809, both *p* < 0.001). The volume of GTV_CTdur_ was 16.74 (2.92–57.13) cm^3^ and exhibited a significant positive correlation with both GTV_PETdur_ (**Figure 1B**) and GTV_DWIdur_ (**Figure 1E**) (*r* = 0.826 and *r* = 0.703, both *p*< 0.001). Similarly, the relative changes in the volume of PET-CT and DW-MRI (∆GTV_PET_, ∆GTV_DWI_) before and during RT demonstrated a significantly positive correlation with that of CT (∆GTV_CT_) before and during RT (*r* = 0.616 and *r* = 0.716, both *p* < 0.001) (**Figure 1**).

### SUV and ADC Values

**Table 2** summarizes the results of SUVs (SUV_max_, SUV_mean_, and SUV_peak_) and ADCs (ADC_mean_ and ADC_min_) performed before and during RT. The differences in SUVs (SUV_max_, SUV_mean_, and SUV_peak_), MTV, TLG, and ADCs (ADC_mean_ and ADC_min_) values as determined on ^18^F-FDG PET-CT and DW-MRI before and during RT were significant (both *p <* 0.001). A trend toward lower SUV and higher ADC was observed during the treatment process.

**Table 2:** Comparison of tumor ADC and SUV values before and during radiotherapy.

Parameters	PET(DWI)_pre_	PET(DWI)_dur_	∆[PET(DWI)_dur_-PET(DWI)_pre_]	*Z-*value	*p-*value
**SUV_max_**	14.10 [3.02–22.94]	8.21 [1.74–13.75]	5.25 [−5.21–15.45]	−4.394	<0.001
**SUV_mean_**	6.87 [1.32–12.75]	4.93 [2.14–8.04]	2.29 [−2.63–9.70]	−4.133	<0.001
**SUV_peak_**	9.98 [3.57–19.34]	5.97 [2.03–11.10]	4.42 [0.03–10.53]	−4.937	<0.001
**MTV**	13.77 [1.07–90.50]	10.3 [1.74–54.38]	4.23 [−5.10–62.51]	−3.571	<0.001
**TLG**	94.7 [4.49–833.10]	35.13 [9.72–285.20]	47.62 [−12.44–721.10]	−4.600	<0.001
$\text{AD}{\text{C}}_{\min}^{\text{a}}$	0.51 [0.30–1.04]	0.79 [0.22–2.09]	0.33 [−0.40–1.19]	−3.909	<0.001
$\text{AD}{\text{C}}_{\text{mean}}^{\text{b}}$	1.30 [0.92–1.83]	2.28 [1.13–4.24]	0.9 [0.21–2.51]	−4.937	<0.001

a ADC_min_ and ADC_mean_ are expressed in 10^–3^mm^2^/s.

PET, positron emission tomography; DWI, diffusion-weighted imaging; SUV_max_, maximum standardized uptake value; SUV_mean_, mean standardized uptake value; SUV_peak_, peak standardized uptake value; MTV, metabolic tumor volume; TLG, total lesion glycolysis; ADC_min_, the minimum apparent diffusion coefficient; ADC_mean_, the mean apparent diffusion coefficient.

### Correlation of ADC and SUV Values

The tumor ADC and SUV values before and during RT showed negligible correlations (pre-RT: SUV_max_ vs. ADC_min_ *r* = −0.322, *p* = 0.072; SUV_max_ vs. ADC_mean_ *r* = −0.217, *p* = 0.232; SUV_mean_ vs. ADC_min_ *r* = −0.258, *p* = 0.153; SUV_mean_ vs. ADC_mean_ *r* = −0.256, *p* = 0.158; dur-RT: SUV_max_ vs. ADC_min_ *r* = −0.133, *p* = 0.496; SUV_max_ vs. ADC_mean_ *r* = −0.133, *p* = 0.496; SUV_mean_ vs. ADC_min_ *r* = −0.013, *p* = 0.944; SUV_mean_ vs. ADC_mean_ *r* = −0.121, *p* = 0.510). There was no correlation between ∆SUV values (∆SUV_max_, ∆SUV_mean_, and ∆SUV_peak_) and ∆ADC values (∆ADC_min_ and ∆ADC_mean_) (*p* = 0.238−0.854) (**Table 3**).

**Table 3:** Correlation analysis of relative changes in SUV and ADC values before and during radiotherapy.

	Parameters	∆SUV_max_	∆ADC_mean_	∆SUV_peak_	∆MTV	∆TLG	∆ADC_min_	∆ADC_mean_
∆**SUV_max_**	*r*-value	1	0.894	0.833	0.154	0.622	−0.196	−0.087
	*p*-value		<0.001	<0.001	0.399	<0.001	0.238	0.635
∆**SUV_mean_**	*r*-value		1	0.870	0.236	0.715	−0.179	−0.035
	*p*-value			<0.001	0.193	<0.001	0.327	0.848
∆**SUV_peak_**	*r*-value			1	0.316	0.784	−0.139	−0.034
	*p*-value				0.078	<0.001	0.448	0.854
∆**MTV**	*r*-value				1	0.739	−0.253	−0.238
	*p*-value					<0.001	0.163	0.190
∆**TLG**	*r*-value					1	−0.286	−0.163
	*p*-value						0.112	0.372
∆**ADC_min_**	*r*-value						1	0.179
	*p*-value							0.327
∆**ADCmean**	*r*-value							1
	*p*-value							

SUV_max_, maximum standardized uptake value; SUV_mean_, mean standardized uptake value; SUV_peak_, peak standardized uptake value; MTV, metabolic tumor volume; TLG, total lesion glycolysis; ADC_min_, the minimum apparent diffusion coefficient; ADC_mean_, the mean apparent diffusion coefficient.

### Associations of SUVs and ADCs With Clinical Prognostic Factors

**Table 4** shows associations of SUVs and ADCs with clinical T-stage and longitudinal length of GTVs. The SUVs (SUV_max_, SUV_mean_, and SUV_peak_), MTV, and TLG pre-RT and its relative changes between pre-RT and after 15 fractions of RT were significantly higher in stages T3–4 than in stage T2 and in the group with a longitudinal length of GTVs ≥4 cm than <4 cm (*p* = 0.000−0.041). The ADC_min_ dur-RT and its relative changes between pre-RT and after 15 fractions of RT were significantly lower in the group with a longitudinal length of GTVs ≥4 cm than <4 cm, but these were not significantly associated with clinical T-stage (**Table 4**).

**Table 4:** Associations of SUVs and ADCs with clinical T-stage and longitudinal length of GTVs.

Parameters	Clinical T-stage			Longitudinal length of GTVs			
	cT2 (*n* = 11)	≥cT3 (*n* = 21)	*p-*value	<4 cm (*n* = 15)	≥4 cm (*n* = 17)	*p-*value	
**Preradiotherapy PET(DWI)**							
**SUV_max_**	7.67 [3.02–15.67]	16.89 [10.75–22.94]	0.001	10.75 [3.02–19.91]	17.23 [11.7–22.94]		0.000
**SUV_mean_**	4.02 [1.32–8.83]	9.21 [5.33–12.75]	0.000	5.8 [1.32–12.75]	9.45 [5.33–12.63]		0.002
**SUV_peak_**	5.86 [3.57–11.31]	12.65 [8.69–19.34]	0.000	7.16 [3.57–11.31]	13.73 [9.54–19.34]		0.000
**MTV**	7.99 [1.07–14.67]	18.86 [2.11–90.50]	0.000	8.26 [1.07–14.67]	19.21 [8.52–90.50]		0.000
**TLG**	24.89 [4.49–72.94]	144.86 [26.9–833.1]	0.000	26.90 [4.49–88.8]	196.71 [82.3–833.1]		0.000
**ADC_min_**	0.55 [0.30–1.04]	0.39 [0.31–0.63]	0.074	0.57 [0.30–1.04]	0.46 [0.31–0.63]		0.079
**ADC_mean_**	1.31 [0.92–1.52]	1.27 [1.07–1.83]	0.706	1.3 [1.07–1.83]	1.3 [0.92–1.64]		0.584
**Dur**-**radiotherapy PET(DWI)**							
**SUV_max_**	4.91 [1.74–13.69]	10.46 [3.76–13.75]	0.025	6.68 [1.74–13.69]	10.46 [3.76–13.75]		0.011
**SUV_mean_**	3.31 [2.14–8.04]	5.10 [2.43–7.33]	0.088	3.43 [2.14–8.04]	5.14 [2.43–7.33]		0.076
**SUV_peak_**	3.07 [2.03–6.53]	7.56 [3.20–11.10]	0.000	3.33 [2.03–6.53]	7.79 [3.2–11.10]		0.000
**MTV**	4.45 [1.74–15.55]	10.85 [2.13–54.38]	0.077	4.74 [1.74–15.55]	11.81 [2.13–54.38]		0.012
**TLG**	24.44 [9.72–45.56]	53.84 [10.18–285.20]	0.006	24.44 [9.72–45.56]	62.41 [14.1–285.20]		0.001
**ADC_min_**	0.92 [0.27–2.09]	0.67 [0.22–1.19]	0.131	1.03 [0.27–2.09]	0.67 [0.22–1.19]		0.010
**ADC_mean_**	2.42 [1.66–4.24]	2.23 [1.13–2.73]	0.126	2.37 [1.66–4.24]	2.23 [1.13–2.73]		0.355
**Relative change from preradiotherapy PET(DWI)to dur**-**radiotherapy PET(DWI)**							
△**SUV_max_**	1.59 [−5.21–11.56]	6.74 [0.21–15.45]	0.010	1.97 [−5.21–15.45]	6.77 [0.87–14.13]		0.013
△**SUV_mean_**	0.44 [−2.63–4.07]	2.90 [−0.33–9.7]	0.002	0.71 [−2.63–9.7]	3.08 [−0.33–7.02]		0.005
△**SUV_peak_**	3.11 [0.75–4.78]	5.36 [0.03–10.53]	0.004	3.47 [0.75–5.36]	5.72 [0.03–10.53]		0.003
△**MTV**	−0.20 [−5.10–5.18]	7.84 [−4.45–62.51]	0.005	1.81 [−5.1–5.18]	9.48 [−4.45–62.51]		0.001
△**TLG**	9.56 [−12.44–48.7]	88.96 [4.06–721.10]	0.000	16.72 [−12.44–48.70]	123.98 [4.06–721.10]		0.000
△**ADC_min_**	0.34 [−0.40–1.19]	0.30 [−0.37–0.56]	0.525	0.38 [−0.40–1.19]	0.20 [−0.37–0.56]		0.041
△**ADC_mean_**	0.99 [0.23–2.51]	0.75 [0.21–1.42]	0.132	0.99 [0.23–2.51]	0.87 [0.21–1.46]		0.45

SUV_max_, maximum standardized uptake value; SUV_mean_, mean standardized uptake value; SUVpeak, peak standardized uptake value; MTV, metabolic tumor volume; TLG, total lesion glycolysis; ADC_min_, the minimum apparent diffusion coefficient; ADC_mean_, the mean apparent diffusion coefficient.

### Differences in Volumes, CI, and DI

The target volumes defined using PET-CT and DW-MRI before and during RT are listed in **Table 5**. The median volume variabilities between GTV_PETpre_ and GTV_DWIpre_ and between GTV_PETdur_ and GTV_DWIdur_ were significant (*p* =0.026 and 0.000, respectively). Significant differences were observed between the CI of GTV_PETpre_ to GTV_DWIpre_ (0.47 [0.20−0.77]) and GTV_PETdur_ to GTV_DWIdur_ (0.29 [0.11−0.48]) (*Z* = −4.750, *p* < 0.001). Meanwhile, the DI of GTV_PETpre_ in GTV_DWIpre_ (0.63 [0.24−2.60]) was significantly larger than that of GTV_PETdur_ in GTV_DWIdur_ (0.38 [0.11−0.92]) (*Z* = −4.675, *p* < 0.001).

**Table 5:** Summary of the volume of GTVs contoured using PET-CT and DW-MRI before and during radiotherapy.

Modality	Target volumes			*Z*-value	*p*-value
	Median	Range			
		Min	Max		
**GTV_PETpre_**	13.77	1.07	90.50	−2.225	0.026
**GTV_DWIpre_**	12.16	4.74	46.86
**GTV_PETdur_**	10.30	1.74	54.38	−3.815	0.000
**GTV_DWIdur_**	5.54	1.65	19.30

### Tumor Maximum Diameter/Volume Shrinkage Rate

**Figure 2:**
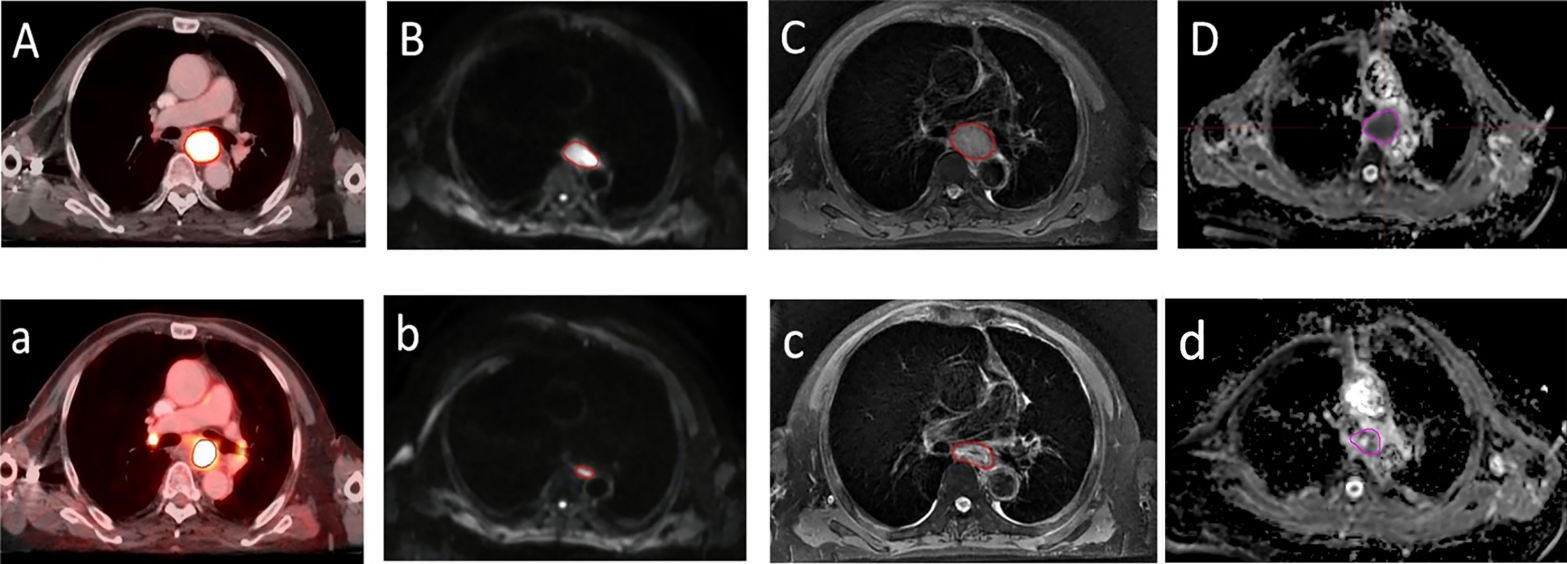
A transversal diagram of gross target volumes on esophageal cancer with high uptake on ^18^F fluorodeoxyglucose positron emission tomography/computed tomography fused images. **(A,a)** Corresponding tumor on T_2_-weighted imaging **(C,c)** with a high signal on diffusion weighted magnetic resonance imaging (b=600 s/mm^2^) **(B,b)** and corresponding apparent diffusion coefficient map **(D,d)** with restricted diffusion at the location of the tumor before radiotherapy **(A–D)** and during radiotherapy **(a–d)**.

There was no significant difference between LD_PETpre_ and LD_DWIpre_ and between LD_PETdur_ and LD_DWIdur_ (median 2.85 cm [1.48−6.31] vs. 2.92 cm [1.89−5.33], median 2.36 cm [1.47−4.79] vs. 2.22 cm [1.35−3.47], *Z* = −1.169 and −1.187, *p* = 0.243 and 0.235, respectively). There was a significant positive correlation between LD_PETpre_ and LD_DWIpre_ and between VRR_PET_ and VRR_DWI_ (*r* = 0.631 and 0.547, *p =* 0.000 and 0.001, respectively). **△**LD_DWI_ (24%) and VRR_DWI_ (60%) based on DWI during RT were significantly greater than the corresponding PET-based **△**LD_PET_ (14%) and VRR_PET_ (41%) (*Z* = −2.393 and −3.758, *p* = 0.017 and 0.000, respectively) (**Figure 2**).

## Discussion

In this single-center prospective study, comparisons of the spatial overlap and functional markers derived from ^18^F-FDG PET-CT and DW-MRI before and during RT based on the medium of CT imaging were evaluated. The results of the current study show that it is feasible to select boosting of high ^18^F-FDG uptake zones within the GTV based on FDG PET-CT for definition of the volume in need of dose escalation (4, 5, 23). However, owing to exorbitant costs and physical burden to patients undergoing repeated PET procedures, MRI-guided Linear Accelerator (MRI-LINAC) with online MR-guided adaptive radiotherapy (MRgRT) in EC has been widely applied (24). Furthermore, ADC measurements on DW-MRI have potential for prediction of response to treatment in esophageal cancer patients, especially the relative ADC increase during and after treatment showed a trend towards a larger increase of ADC in good responders compared with poor responders (16). Hence, we aimed to explore whether the high signal area derived from DW-MRI can be used as tool for an individualized definition of the volume in need of dose escalation for EC. To our knowledge, this is the first prospective trial assessing the geometrical differences and metabolic parameters between two imaging modalities in the reirradiation treatment planning for esophageal squamous cancer.

Currently, the method of gradient-based algorithm (PET_Edge_) has been found to correspond better to pathological specimens than manual or relative threshold-based methods (19). However, no single PET-based segmentation algorithm has yet performed better than manual CT delineation alone (16), implying that PET-guided adaptive radiotherapy was insufficient for clinical decision-making. Till date, CT imaging remains the gold standard for GTV delineation and treatment planning evaluation. On this basis, our data suggest that the volume of GTV_CT_ exhibited a significant positive correlation with that of GTV_PET_ and GTV_DWI_.

Based on our analysis, the results showed that the differences in SUV and ADC values before and during RT were significant, consistent with the findings of more recent studies (25, 26). There is also clear evidence that higher SUV and lower ADC values are associated with a higher histological grade and aggressiveness (17, 27). With high cellular density and enhanced glucose metabolism, malignant tumors generally exhibit low ADCs and high SUVs. Several studies indicate that the change in tumor ^18^F-FDG uptake for EC seemed highly predictive for assessing response during and after treatment (7, 28). Another recent study by Aerts et al. (29) has demonstrated that the recurrent areas within the tumor after therapy largely corresponded with the high FDG uptake area of the pretreatment PET scan. As a result, selective boosting of high ^18^F-FDG uptake zones within the tumor for radioresistance has been suggested. However, part of the limitations of PET can be attributed to the fact that no consensus for the accurate segmentation algorithm is recommended. Meanwhile, DW-MRI is emerging as an advanced imaging technique with noninvasive, well-tolerated, and excellent soft-tissue contrast features for diagnosing EC. ADC value is the most common DWI-derived imaging biomarker with broad clinical applications. More importantly, recent exploratory studies have shown that changes in ADC appears to provide valuable information on the prediction and assessment of treatment response early after RT (7, 16, 17). Consequently, regions of restricted diffusion may serve as a surrogate for active tumor tissue. DW-MRI may be a technically and clinically available alternative to PET-CT for an individualized definition of the volume in need of dose escalation for EC.

Concerning the possible correlation between ADCs and SUVs for the prediction of survival or evaluation of response to treatment in EC, our current data suggest that the tumor ADCs and SUVs before and during RT showed negligible correlations. Similar to our findings, previous studies also found no significant correlations between pretreatment SUVs and ADCs (30–32). Our results also revealed that pretreatment SUVs, MTV, and TLG were significantly higher in tumor stage ≥ T_3_ than in tumor stage < T_2_, while ADC_min_ values has not yet been found to correlate with the clinical T-stage, indicating the effect of tumor load on ^18^F-FDG metabolism, consistent with the literature (8, 33). These results suggest that enhanced glucose metabolism as measured on FDG PET and restricted water diffusion as measured using ADC represent independent biological properties and refer to different aspects of tumor pathophysiology. This is likely owing to the variety of pathogenic mechanisms because of which elevated ^18^F-FDG uptake was detected in the sites of glucose metabolism and active inflammation, while no significant changes in cell density were detected in the activity of inflammatory cells (28, 33, 34). Additionally, necrosis and liquefaciens induced by radiation can impede movement of water molecules, leading to increased ADC values. Finally, it is likely that the timing and distribution of decreased glucose metabolism and cellular density are asynchronous and inconsistent.

In addition, we evaluated the difference in matching and inclusion relation between the GTVs derived from PET-CT and DW-MRI simulation before and during RT. Our results indicate that the CI of GTV_PETpre_ to GTV_DWIpre_ was significantly larger than that of GTV_PETdur_ to GTV_DWIdur_. Meanwhile, a significant difference was observed between the DI of GTV_PETpre_ in GTV_DWIpre_ and GTV_PETdur_ in GTV_DWIdur_. Our study results are also consistent with the findings of Popp et al. (34) and Houweling et al. (35), who showed that the GTV of restricted diffusion on ADC overlapped only partially with that of increased glucose uptake for reirradiation treatment planning, suggesting that there were great mismatches between the regions of residual high FDG uptake based on PET-CT and the areas of residual high signal based on DW-MRI. Moreover, given the data from our study, the rate of tumor maximum diameter/volume regression based on DW-MRI during RT is significantly faster than that based on PET-CT. This suggests that the regions of high cellularity may not cover the entire biologically active tumor, agreeing with earlier studies comparing DWI and PET in reirradiation of recurrent primary brain tumors (34, 35).

Some limitations of the current study must be considered. A potential disadvantage of EPI-DWI is that the technique is prone to artifact contamination caused by variations in magnetic susceptibility (36). To minimize this, the study excluded some cases with a small volume and severe image distortions. Additionally, The magnitude of the geometric distortion scales with magnetic field strength (37). Considering that the use of 3.0 T MRI was applied, the limitation of the geometric distortion caused by high field strength must be considered. Owing to the partial-volume and pseudo-diffusion effect caused by tumor vascular permeability and microcirculation perfusion, tumor shrinkage upon initiation of treatment may lead to underestimation of the FDG uptake observed on midtreatment imaging modalities and a consequent overestimation of the change in parameters such as the SUV. Further dosimetric investigations are necessary to evaluate whether it is safe to select boosting of the high signal areas within the tumor based on DW-MRI for definition of the volume in need of dose escalation.

## Conclusions

The location of high residual FDG uptake based on ^18^F-FDG PET-CT yielded poorer spatial matching than that of high residual signal based on DW-MRI during RT. Furthermore, tumor ADC and SUV values may play complementary roles as imaging markers for the prediction of patterns of failure and for definition of the volume in need of dose escalation. In addition, the rate of tumor maximum diameter/volume regression based on DW-MRI during RT is significantly faster than that based on PET-CT. Based on the medium of CT images, the volume of GTV_CT_ exhibited a significant positive correlation with that of GTV_PET_ and GTV_DWI_. Under this premise, there are significant differences in spatial position, biometabolic characteristics, and the tumor shrinkage rate for GTVs derived from ^18^F-FDG PET-CT and DW-MRI before and during RT for esophageal squamous cancer. Further studies are needed to determine if DW-MRI will be used as tool for an individualized definition of the volume in need of dose escalation.

## Data Availability Statement

The original contributions presented in the study are included in the article/supplementary material. Further inquiries can be directed to the corresponding authors.

## Ethics Statement

The studies involving human participants were reviewed and approved by the Shandong Tumor Hospital Ethics Committee. The patients/participants provided their written informed consent to participate in this study. Written informed consent was obtained from the individual(s) for the publication of any potentially identifiable images or data included in this article.

## Author Contributions

HL contributed to the study design, the patient enrollment, the data statistics, and analysis and writing of the manuscript. JL and FL participated in the study design and revised the content. YZ and YL contributed to reviewing the delineation. YG and LX made important contributions in collecting the data and revising the content. All authors read and approved the final version of the manuscript.

## Funding

This work was supported by the National Natural Science Foundation of China (grant number 81773287) and Taishan Scholars Program of Shandong Province (grant number ts20190982).

## Conflict of Interest

The authors declare that the research was conducted in the absence of any commercial or financial relationships that could be construed as a potential conflict of interest.

## Publisher’s Note

All claims expressed in this article are solely those of the authors and do not necessarily represent those of their affiliated organizations, or those of the publisher, the editors and the reviewers. Any product that may be evaluated in this article, or claim that may be made by its manufacturer, is not guaranteed or endorsed by the publisher.
